# Metacommunity: the current status of psychiatry and mental healthcare and implications for the future

**DOI:** 10.1192/bji.2024.15

**Published:** 2024-08

**Authors:** George Ikkos, Nick Bouras

**Affiliations:** 1 Consultant Liaison Psychiatrist, Royal National Orthopaedic Hospital, Stanmore, UK. Email: ikkos@doctors.org.uk; 2 Emeritus Professor of Psychiatry, Institute of Psychiatry, Psychology & Neuroscience, King's College London, London, UK

**Keywords:** Community psychiatry, neoliberal political economy, healthcare technology, neuroscience, mental health activism

## Abstract

We review the origins and history of community psychiatry and the challenges posed to it by advancing technology and the neoliberal political economy and society that have prevailed since the 1990s. We summarise both achievements and shortcomings and argue that the term ‘community’ fails to acknowledge the gap between its original ambition and the outcomes of its implementation. We argue that, because of the changes that have taken place, the implementation of community psychiatry's objectives as conceived originally is likely to continue to fail. To sharpen current awareness and thinking and optimise future policy discourse and service strategies we revisit the concept of ‘metacommunity’. This is a historical descriptive label that aims to encapsulate the fundamental transformations that have taken place. These in turn demand of psychiatrists and other mental health providers both more socially critical thinking and mental health activism in the public sphere. Ultimately, beyond both community and metacommunity psychiatry, what is required is a democratic psychiatry.

One billion people worldwide (more than one in eight adults and adolescents) live with ‘mental disorders’, at the cost of nearly US$1 trillion. Despite the massive problem and astounding economic cost, it has been estimated that only 29% of people with psychosis receive mental health services.^1^ The *Lancet* Commission on Global Mental Health and Sustainable Development^1^ and WPA-*Lancet* Psychiatry Commission on the Future of Psychiatry^2^ have set out the challenges that psychiatrists and patients in their care face and advocate community psychiatry as the way forward. We fully embrace the aims and thrust of community psychiatry but after more than five decades of endeavour it has fallen short of its objectives. This is because political economic circumstances have changed worldwide in ways that fundamentally undermine or alter the very idea of community as historically understood.^3^ In other words, the term community now serves more as a nostalgic idealisation than as an illuminating beacon for the future. To develop a more incisive perspective we revisit and update the concept of ‘metacommunity’ psychiatry and mental healthcare (‘metacommunity psychiatry’)^4^ and draw on its implications.

That the need for the concept of metacommunity arises is in large part due to wider political economic, technological and social changes is a key point. Crucially, around the same time as deinsitutionalisation and community care were pressed most vigorously (from the 1990s onwards in the UK), neoliberalism, globalisation and the dominance of international finance had succeeded in undermining the very nature and importance of the welfare state, even the very meaning and power of the ‘national community’ in some countries.^5^ The stripping away of workers’ rights, job insecurity, demand for occupational mobility, waves of immigration and oppressive target-driven learning and working conditions, coupled with grotesque financial inequalities, have fractured the very local communities that were meant to contain and care for those with mental health conditions. Lack of resources and inequity in mental healthcare provision have prevailed.^6,7,8^ But the changes have not been only destructive. They have also been generative of what some call ‘neocommunities’^3^ (p. 3). These are very different from the communities that the pioneers of community psychiatry had in mind. For example, members of immigrant communities from the Global South to the North may feel they belong more to the transnational communities of their respective diasporas than the local communities where their new homes are located.

In Greek ‘meta’ means ‘after’. Thus, metacommunity psychiatry means after community psychiatry. As intended in our formulation, the term ‘metacommunity’ is historical, descriptive of our current state in psychiatry and mental health services. It is not a policy statement nor a political strategy. The use of the term is intended to raise awareness of the fact that the relation between the state, psychiatry and our patients has been transformed fundamentally as a result of specific political economic and technological changes. A second key point of the present article, therefore, is to suggest that, because of this transformation, psychiatrists and other mental health professionals need to complement our collaborative engagement with the state with explicitly more socially critical thinking about the powers that determine real-life outcomes and to adopt a more activist engagement in the public sphere.^9,10^

## Community psychiatry: a reckoning

In those countries that had large psychiatric hospitals, community psychiatry emerged through policies of deinstitutionalisation. The circumstances and motives were complex, including historical, economic and cultural ones. A major impetus came from the tremendous advances in psychopharmacology in the 1950s. At the time, psychiatrists could proclaim confidently that a new dawn had emerged in the treatment of patients, most with severe conditions. As a result they could be returned promptly and safely to be reintegrated back into their communities, or even remain at home throughout their acute crisis and recovery. Of course, the confidence of psychiatrists did not remain unchallenged. Another vital contributor to the imperative of community psychiatry had been the critique of those who came to be known as anti-psychiatrists, later the critical psychiatrists and their fellow travellers too. However, many psychiatrists, anti-psychiatrists and others around them were united on the importance of social factors in the treatment, rehabilitation and secondary prevention of mental health conditions. Quite a remarkable consensus took hold, including across political philosophical and party political lines.

Community psychiatry emerged during the post-Second World War liberal-/social-/Christian-democratic consensus. Whole national populations had faced the war ‘together’ and there was then a commitment to both community and the welfare state. In its implementation, community psychiatry embraced the biopsychosocial model. Although this has served well and has gone some way to meet the above critiques, in practice it has retained the idea of mental disorders as medical disorders invariably to be diagnosed and treated ‘like any other’ and this failed to satisfy many critics. Vindicating some critics, perhaps surprisingly, the former director of the National Institute of Mental Health in the USA has acknowledged the failure of the ‘Decade of the Brain’ (the 1990s) and neuroscience to deliver better outcomes^11^ and increasingly some of the foremost leaders of the profession are concluding that current models of psychopathology ‘do not fit the data’^12^ and that diagnostic systems ‘lack validity’.^13^ Meanwhile, as a result of neoliberal market fundamentalism and the politics and policies associated with it,^14^ newly prevalent phenomena of street homelessness, penal incarceration and trans-institutionalisation of those with mental health conditions have disappointed ambitions. Furthermore, the phenomenon of ‘deaths of despair’^15^ has been described and the prevalence of mental health problems associated with war, dislocation and asylum-seeking is increasing, as is that of eating disorders and other common mental health problems. The USA has been at the forefront of both political economic and technological changes, and data from the *Lancet* Commission on Public Policy and Health in the Trump Era suggest that the country has experienced approximately 900 million excess deaths compared with other G7 countries between the years 1980 and 2018, with a constantly increasing excess over the decades (see Fig. 2 in the Commission's report^16^). All this demands an honest reckoning and recalibration.

The idea of community psychiatry has prevailed in the context of human rights advances and liberalising mental health laws and has helped to focus on the plight of people with severely disabling mental health conditions to develop crisis and home treatment interventions and address issues of stigma. Evidence for the effectiveness of supported employment and housing-first policies has emerged. No longer socially excluded in remote depersonalising institutions, people with mental health conditions began to find their voice and the service user movement has taken hold.^17,18^ Not everything has worked according to plan, however. Recently, Kirkbride et al have summarised increasing evidence for the deleterious effect of structural inequalities on mental health as well as the effectiveness of preventive social approaches.^19^ Conditions for those left behind in institutions deteriorated and scandals recurred. Communities became anxious about mentally ill newcomers in their midst and, goaded by scurrilous tabloid newspaper headlines, have too frequently rejected those discharged from institutions. Especially because of failures of social policies and services since 2008, continuity of care, mental symptom control and physical health have suffered^20^ and the families of those with severely disabling mental health conditions have too often been let down by the quality (or lack) of care and felt overwhelmed by the burden placed on them.^18,21^

## 1979 and the neoliberal and technological challenge to community psychiatry

A pivotal year was 1979:^22^ first, the election of Margaret Thatcher in the UK (and Ronald Regan the year after in the USA) ushered in the drive to market fundamentalism and a new globalisation; second, the award of the Nobel Prize in Physiology or Medicine for the development and commercialisation of the computed tomography (CT) scanner headlined the technology that from now on would turn psychiatry away from social and towards biological priorities; and third, the publication of Jean-François Lyotard's *La Condition Postmoderne* (*The Post-Modern Condition*)^23^ signalled the rise of capital, metrics and management at the expense of feelings, narrative and difference. The introduction of the concept of metacommunity is meant to highlight the transformational significance of these changes in political economy and the contemporaneous rapid advances in clinical and information technology and their impact on psychiatry. Also, there was the later emergence of social media. Online communities are very different from geographical ones, and they offer both entirely new threats and opportunities. For example, gender identity issues have acquired unprecedented visibility and significance.

## Metacommunity psychiatry is now

The concept of metacommunity psychiatry is primarily descriptive and aims to identify and label an era in the history of psychiatry and work out its significance. Metacommunity psychiatry is psychiatry today, i.e. the outcomes of the transformations summarised above. It is psychiatry at a time when the dappled nature of psychopathology has become increasingly clear;^24^ the high prevalence of physical and mental comorbidity has firmly challenged the separation of physical and mental health; and the recognition of autoimmune encephalopathy, albeit arising from neurology, has offered new insights and therapeutic opportunities in relation to cases of acute psychosis that previously remained mysterious and were managed ineffectively under the broad label of schizophrenia.^25^ The number of online communities has exploded. In cyberspace multiple subjectivities and opinion may too often trump evidence, and debates on neurodiversity have both enlightened and confused. Now is when structural inequalities prevail; algorithms, metrics and managers drive clinical action; deaths of despair, homelessness, compulsion and incarceration have been increasing; and, in a move that may have surprised the pioneers of community psychiatry and grates with some of its advocates today, both many psychiatrists and many patients have invested much hope in ketamine and psychedelics.

Demarcating between eras in history is no easier than between psychiatric diagnoses. If the turning point was 1979 and the first shoots of metacommunity could be found in the 1990s, now post COVID-19, with a decisive increase in remote care and the arrival of ‘the age of AI’,^26^ we find ourselves in uncharted territory, full of threats and ambiguities as well as beguiling promises. It may be a coincidence that Facebook renamed itself Meta in 2021, but metacommunity psychiatry is a reality whose era has come!

## The future

Psychiatry in the metacommunity era embraces evidence, clinical effectiveness and robust outcome, and qualitative research to identify the views of patients, carers and staff.^27^ But while community psychiatry advocated the development of mental health services locally,^28^ its metacommunity successor must also take explicitly into consideration the broader sociopolitical, technological and scientific context. In the light of experience in recent decades and emerging debates about so called techno- or neo-feudalism (Box 1), the risk is that the future evolution or demise of neoliberalism may embrace more demagogic, inequitable and authoritarian^29^ rather than liberal and socially inclusive ideals. Although we must continue cooperating with the state, we can no longer assume that working with it will produce better results in the future. Persistent shortcomings in meeting the World Health Organization's Sustainable Development Goals and the increasing evidence of our environmental crisis and its adverse effects on mental health confirm the importance of scepticism. We must adopt a critical stance towards the powers that drive state policies and determine real-life outcomes, one that is evidence-informed but also facilitates activism where this is necessary.^30^ Although one of the key changes between the early years of community psychiatry and today has been a massive demographic shift from young to older people in high-income and some other countries,^31^ if we are to successfully meet the rapidly increasing mental health needs of the global population, we must recapture something of the spirit of critique of the 1960s and challenge the boundaries, even break out beyond the limits of professional neutrality if necessary. At the same time, we must commit firmly to clinical pluralism and compassionate and relational care.^32^

Box 1 Techno- or neo-feudalism

In political economic discourse, techno- or neo-feudalism refer to the proposition that the world economy is evolving towards social relations between a small powerful elite and the bulk of the population that resemble those of the nobility and serfs in feudal societies. Those advocating such a view attribute this evolution to the erosion of civil and workers’ rights and social injustices arising out of neoliberal economics, digital technologies, copyright laws and dominance of the sector by a small number of companies that are increasing inequalities in wealth and power (For an informative debate on relevant issues see: Morozov E. Techno-feudalism? *New Left Rev* 2022; **133/134**: 89–12; Durand C. Scouting capital's frontiers. *New Left Rev* 2022; **136**: 29–39; Rikap C. Capitalism as usual? *New Left Rev* 2023; **139**: 145–60.)

## A final word

The era of metacommunity psychiatry described in this article is mostly relevant to high-income countries, but as low- and middle-income countries will grapple with the ‘mental health gap’ in an accelerating trajectory we anticipate it will become increasingly relevant there also, not least because of the exploding numbers of their young people. Ultimately, we should aim beyond metacommunity towards a democratic psychiatry which, both in the clinic and beyond, actively challenges structural inequalities and secures prevention, social inclusion and equitable access to high-quality mental healthcare.

## Data Availability

Data availability is not applicable to this article as no new data were created or analysed in this study.
